# Clinical evaluation of severe neonatal Hyperbilirubinaemia in a resource-limited setting: a 4-year longitudinal study in south-East Nigeria

**DOI:** 10.1186/s12887-018-1174-z

**Published:** 2018-06-23

**Authors:** Chidiebere D. I. Osuorah, Uchenna Ekwochi, Isaac N. Asinobi

**Affiliations:** 1 Child Survival Unit, Medical Research Council UK, The Gambia Unit, Fajara, Banjul, Gambia; 2 Department of Paediatrics, Enugu State University of Science and Technology, Enugu, Enugu State Nigeria

**Keywords:** Newborns, Severe hyperbilirubinaemia, Causes, Clinical features, Complications, Enugu

## Abstract

**Background:**

Neonatal hyperbilirubinaemia is one of the commonest causes of hospital visit in the neonatal period. When severe, it is a leading cause of irreversible neurological and musculoskeletal disability. Prompt recognition and timely interventions are imperative for a drastic reduction in complications associated with severe hyperbilirubinaemia in newborns.

**Methods:**

We report a 4-year descriptive and longitudinal study to determine the causes, clinical presentations and long-term outcomes in newborns admitted for severe neonatal jaundice. Methods: Newborns admitted and managed for severe neonatal jaundice at the Enugu State University Teaching Hospital during a 4-year period were enrolled and followed up for 2 years.

**Results:**

A total of 1920 newborns were admitted during the study period and 48 were managed for severe hyperbilirubinaemia giving an in-hospital incidence rate of 25 (95% CI 18–32) per 1000 admitted newborns. The mean age of onset was 3.4 ± 0.5 days (range 1–8 days) and hospital presentation from time of first notice was 4.3 ± 0.4 days (range 1–9 days). The total and unconjugated admission serum bilirubin ranged from 7.1 to 71.1 (mean 26 ± 2.5 mg/dl) and 4.2 to 46.3 mg/dl (mean 18.3 ± 9.2) respectively. Earliest sign of severe hyperbilirubinaemia in newborns were: refusal to suck (15.2%) and depressed primitive reflexes (24.5%) while the commonest signs included high pitch cry (11.9%), convulsion and stiffness (6.9%) and vomiting (6.3%) in addition to the former signs. The major causes of severe hyperbilirubinaemia were idiopathic (33.3%), sepsis (35.3%), ABO incompatibility (17.6%) and glucose-6-phosphate dehydrogenase (G6PD) deficiency (11.8%). Long-term sequelae on follow-up included delayed developmental milestone attainment, postural deformities, visual and seizure disorders.

**Conclusions:**

There is urgent need for continued education for mothers, families and healthcare workers on the danger newborns with jaundice could face if not brought early to the hospital for timely diagnosis and management.

## Background

Neonatal Jaundice typically results from the deposition of unconjugated bilirubin pigment in the conjunctiva, skin and mucus membranes when there is excessive amount of bilirubin in blood. Hyperbilirubinaemia is defined as a total serum bilirubin level above 5 mg/dL (86 μmol per L) [1]. It is by far the most common reason for hospital presentation in the neonatal follow up clinic [2]. Although majority of newborns that have clinical jaundice in the first week of life recovers without treatment, some cases of hyperbilirubinaemia can however be serious and if not well managed, could results to severe morbidity and mortality [3]. Neonatal hyperbilirubinaemia is considered pathologic if it presents within the first 24 h after birth, the total serum bilirubin level rises by more than 5 mg/dL (85 μmol/L) per day or if the total bilirubin level is higher than 20 mg/dL (340 μmol/L) in term newborns or lower in term newborns with signs and symptoms suggestive of serious illness [1]. Common risk factors for severe hyperbilirubinaemia includes fetal-maternal blood group incompatibility, prematurity, glucose-6-phosphate dehydrogenase deficiency, hepatic diseases and septicaemia [4]. In order to prevent the potential and irreversible complications of severe hyperbilirubinaemia, exchange transfusion which is the most rapid method of lowering serum bilirubin concentrations is used when serum bilirubin reaches critical level and in other cases when serum bilirubin rises despite intensive phototherapy. This descriptive study conducted in the Enugu State University Teaching Hospital (ESUTH) over a 4-year period assessed the incidence, causes and clinical features of severe hyperbilirubinaemia in newborns after birth and followed them up for 2 years for possible complications after discharge from the hospital. It is hoped that findings from this study would aid clinicians in case identification and prompt management of newborns with risk factors for severe hyperbilirubinaemia.

## Methods

### Study area and site

This was a prospective study carried out in the Neonatal Intensive Care Unit (NICU) of Enugu State University Teaching Hospital (ESUTH). The site is a tertiary health facility that offers specialized medical services and serves as a referral centre to private, general, mission hospitals and other delivery homes within Enugu and the neighbouring states. The NICU offers 24-h services for sick babies born within and outside the hospital within their first 28 days of life. The NICU is manned by consultant neonatologists and resident doctors who are specialists in Paediatrics with further sub-specialist training in neonatology.

### Enrolment of newborns into the study

This study was conducted over a period of four years between January 2013 and January 2017. Newborns with severe hyperbilirubinaemia were consecutively enrolled after obtaining an informed consent from their mothers or caregivers. Presence of clinical features that are related to hyperbilirubinaemia and its complications were documented. Results of certain baseline investigations done to ascertain the level and causes of hyperbilirubinaemia were also documented. These include serum bilirubin level, random blood sugar, glucose-6-phosphate dehydrogenase (G6PD) status, blood group, blood culture etc. Other data obtained included age of newborn at onset of jaundice, time of presentation, the number of exchange blood transfusions (EBT) done (i.e. single or double) and the outcome of the index admission (i.e. alive, dead, and left against medical advice). Those that survived were followed up for 2 years in the post-natal clinic. At each visit, they were reviewed to ascertain presence of deficits in milestone development such as motor, postural, visual, hearing and others. Visual and hearing examination was done using clinical assessment methods such as paediatrics visual chart and sound effects. Where abnormalities are noted, further assessment by an audiologist and eye specialist was sought appropriately. Care-givers of newborns that could not present for follow up were contacted on a 3-monthly basis via phones calls. During the call, information on developmental milestones and date attained were explored. They were also asked for presence of postural, visual, hearing and any other concerns they might have regarding their child’s growth and development. Where abnormalities were encountered, care-givers were requested to bring the child to the outpatient clinic for further evaluation.

### Measures

All aetiologies of jaundice beyond physiologic and breastfeeding or breast milk jaundice are considered pathologic. Features of pathologic jaundice include the appearance of jaundice within 24 h after birth, a total serum bilirubin level higher than 15 mg/dl (256 μmol/L) in preterm newborns and 20 mg/dl (340 μmol/L) in term babies. Others include rise of unconjugated bilirubin by ≥5 mg/dl (85 μmol/L) in 24 h, prolonged jaundice, elevation of the serum conjugated bilirubin level to ≥2 mg/dl (34 μmol/L) and jaundice with evidence of underlying illnesses such as haemolytic conditions, sepsis, liver pathology etc.

### Overview of management of severe hyperbilirubinaemia in NICU of ESUTH

Neonatal jaundice occurring after the 2nd day of life in otherwise healthy babies without other symptoms is sent for urgent serum bilirubin estimation to guide further clinical action. As part of the unit protocols, immediate admission is indicated in jaundice occurring in the 1st of life, levels indicative of pathological jaundice (see above), in preterm, in all sick babies and those with identified risk for bilirubin encephalopathy.

After admission, a thorough history is taken documenting the onset and progress of jaundice, duration, gestational age of the baby at delivery and associated symptoms that signifies imminent risk of encephalopathy such as vomiting, refusal to suckle, weakness, abnormal movements, shrill cry, abnormal breathing etc.

A comprehensive physical examination is also done noting the anatomical level of the jaundice, activity of baby, presence of pallor, vital signs and thorough neurological assessment noting the posture, movement, cry, muscle tone and status of the primitive reflexes. Concurrently, basic laboratory investigations to estimate bilirubin level and identify possible causes are done. These tests include; serum bilirubin level (total and direct), haemoglobin level, random blood sugar, maternal and baby’s blood group and rhesus status, G6PD status for males, blood film for malaria parasite and size/shape of the erythrocyte. Further investigations such as blood culture, serum protein coomb’s test, serum electrolyte, urine and stool analysis, abdominal ultrasonography and blood gas are also ordered based on the findings in the history, physical examinations and initial laboratory tests.

**Fig. 1:**
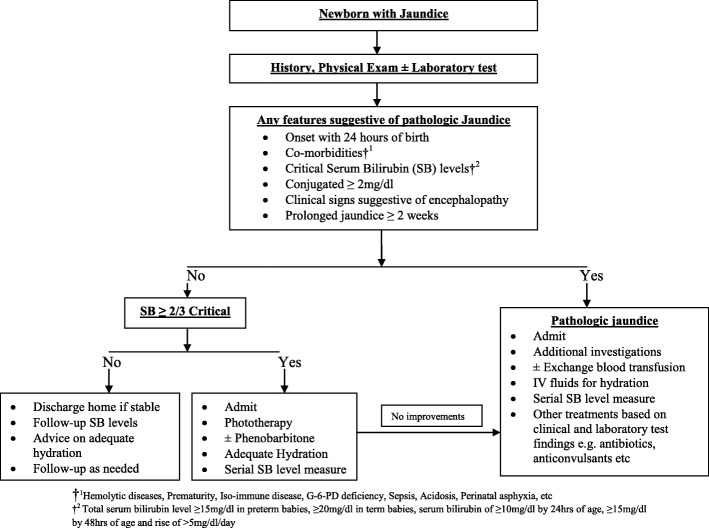
Evaluation of Newborns with Jaundice in Enugu State University Teaching Hospital

Generally, three modalities of treatment are available in our centre and these include; i*)* Pharmacotherapy using phenobarbitone which increases hepatic uptake and metabolism of bilirubin and usually indicated mainly in preterm babies as adjunct therapy in combination with other treatment modalities. ii*)* Phototherapy for serum bilirubin up to 2/3rd of the critical level for exchange blood transfusion and iii*)* Exchange blood transfusion (EBT) is the treatment modality of choice for severe hyperbilirubinaemia in cases where serum bilirubin level reaches up to ≥15 mg/dl in preterm babies; ≥ 20 mg/dl in term babies; and a rise of ≥5 mg/dl in 24 h. However, presence of risk factors for bilirubin encephalopathy like acidosis, sepsis, abnormal neurological findings necessitates EBT even at lower serum bilirubin levels. Most times, combination of these treatment modalities is employed except in cases of conjugated hyperbilirubinaemia where phototherapy is avoided because of the risk of the so called 'Bronze Baby Syndrome' See Fig. 1.

### Data entry and analysis

The above measures were documented at presentation in the relevant sections of the questionnaire and subsequently transferred into a Microsoft Excel Sheet. Distribution of the measures were categorized into sub-variables and reported in percentages. Enrollees with significant missing information were excluded from the data analysis. Data were analysed using IBM® SPSS version 18.0 (SPSS Inc., Chicago, IL).

## Results

### Characteristics of newborns with severe hyperbilirubinaemia

Table 1 shows the main characteristics of newborns enrolled for this study. Of the 1920 newborns admitted to the NICU during the study period, 48 were managed for severe hyperbilirubinaemia giving an in-hospital incidence rate of 25 (95% CI 18–32) per 1000 newborns admitted to the unit. Fifteen (31.2%) of these newborns were delivered within ESUTH while the remainder (68.8%) were delivered outside and referred to the hospital. About two-third of the surveyed newborns were male and term deliveries. Twenty (44.4%) were delivered with low birth weight (< 2.5 kg) and double exchange was the modality of EBT used in the management of severe hyperbilirubinaemia in most (73.9%) cases. Table 2 summaries the baseline parameters of newborns admitted for severe hyperbilirubinaemia during the study period.

**Table 1:** Demographic characteristics of newborns with severe hyperbilirubinaemia admitted to the Enugu state university teaching hospital

S/N	Characteristic	Variable	Number	Percentage
			n	%
1	Gender *(n = 48)*	Male	30	62.5
		Female	18	37.5
2	Birth weight (Kg) *(n = 45)*	< 2.5 kg	20	44.4
		≥ 2.5 kg	25	55.6
3	Gestational Age (Weeks) *(n = 45)*	Term (≥ 37)	33	68.8
		Pre-term (< 37)	15	31.2
4	Place of birth *(n = 48)*	Inborn	15	31.2
		Outborn	33	68.8
5	Number of EBT^a1^ *(n = 46)*	Single	12	26.1
		Double	34	73.9
6	Outcome in Hospital *(n = 48)*	Alive	40	83.3
		Dead	7	14.5
		LAMA^a2^	1	2.2

^a1^EBT exchange blood transfusion

^a2^Left against Medical Advice

**Table 2:** Baseline parameters of newborns admitted with severe hyperbilirubinaemia in Enugu state university teaching hospital

S/N	Parameters	Mean ± SD	SE	Min value	Max value	Range
1	Birth weight (Kg)	2.7 ± 0.9	0.1	1.2	4.9	3.7
2	Age at onset (days)	3.4 ± 0.5	0.4	1.0	8.0	7.0
3	Time of presentation (days)	4.3 ± 0.4	0.4	1.0	9.0	8.0
4	Total serum bilirubin (g/dl)	26 ± 2.5	2.0	7.1	71.1	64.0
5	Unconjugated serum bilirubin	18.3 ± 9.2	1.7	4.2	46.3	42.1
6	Conjugated serum bilirubin	8.4 ± 8.1	1.5	0.5	25.1	24.6
7	Random blood sugar (mmol/L)	132.7 ± 83.8	16.4	48	450	402

SD for Standard deviation and SE for Standard error

### Causes and clinical features of severe hyperbilirubinaemia in newborns

Table 3 recapitulates possible causes and clinical features of severe hyperbilirubinaemia in newborns surveyed. In over two-third of newborns (33.3%), no aetiology was apparent after both clinical and laboratory evaluation. Sepsis was a co-morbidity in 35.3% of these newborns while G6PD deficiency was the apparent cause in approximately 11.8% of the cases. Of note, 50% (3/6) of the infants with G6PD deficiency used camphor prior to onset of severe hyperbilirubinaeia.

**Table 3:** Causes and clinical features of hyperbilirubinaemia in newborns enrolled in Enugu state university teaching hospital

Parameter	Number n^a1^	Percentage %
Causes		
ABO incompatibility	9	17.6
G6PD deficiency^a2^	6	11.8
Sepsis	18	35.3
Rhesus incompatibility	1	2.0
No aetiology seen	17	33.3
Clinical features		
Fever	27	17.0
Convulsion	11	6.9
Refusal to suck	24	15.2
High pitch cry (shrill cry)	19	11.9
Vomiting	10	6.3
Neck retraction	7	4.4
Floppiness (hypotonic)	4	2.5
Stiffness (hypertonic)	11	6.9
Depressed reflexes	39	24.5
Death (while still on admission)	7	4.4

^a1^Multiple causes were noted in some newborns

^a2^G6PD was only tested in male newborns surveyed

Other probable causes were Rhesus incompatibility (2%) and ABO incompatibility seen between nine mother-infant pairs (17.6%). Blood groups of mothers included [O+] 14 (68.5%), [O-] 1 (4.5%), [A+] 1 (4.5%), [B+] 4 (18.2%) and [AB+] 2 (9.1%) while those of newborns included [O+] 13 (48%), [A+] 6 (24%) and [B+] 6 (24%).

Fever (17%), refusal to suck (15.2%) and depressed primitive reflexes (24.5%) were the commonest clinical manifestations in newborns with severe hyperbilirubinaemia. Of the depressed reflexes seen, suckling was the most common affected 15/39 (38.5%). Other affected reflexes included Moro’s reflex 9/39 (23.1), lateral spinal reflex 6/39 and grasp reflex 3/39 (7.7%). There was global areflexia in 10 (20.8%) newborns. Seven of the newborns managed for severe hyperbilirubinaemia died while still on admission giving a case fatality of 14.5% and one was discharged on parent’s request against medical advice (Table 1).

### Long-term complications in newborns with severe hyperbilirubinaemia

Newborns managed for severe hyperbilirubinaemia were followed up for approximately 2 years after discharged from the hospital. Over half (25) were lost to follow up due inability to reach parent on phone and/or refusal to attend follow up clinic. Of the 23 remaining newborns successfully followed up, 10 had motor developmental milestone delays which included attainment of neck control between 6 and 12 month, crawling after 1 year, sitting with support at 2 years and walking without support at 2 years. One child had not achieved neck control at the time of follow-up by the age of 2 years. Similarly, six of these children had postural deformity while 4 died before their second birthday (i.e. at 2, 7, 18 and 21 months). Other complications encountered on follow up included visual impairment in one child and seizure disorder in two children. Table 4 shows a cross-tabulation of the complications encountered and some selected demographic characteristics.

**Table 4:** Long-term complication in newborns with severe hyperbilirubinaemia in Enugu state university teaching hospital

S/N	Characteristic	Variable	Motor delays	Postural deformities	Death
			*n* = 10	*n* = 6	*n* = 4
1	Gender	Male	5 (50.0)	4 (66.7)	1 (25.0)
		Female	5 (50.0)	2 (33.3)	3 (75.0)
2	Birth weight (Kg)	< 2.5 kg	4 (40.0)	2 (33.3)	3 (75.0)
		≥ 2.5 kg	6 (60.0)	4 (66.7)	1 (25.0)
3	Gestational Age (Weeks)	Term (≥ 37)	7 (70.0)	5 (83.3)	2 (50.0)
		Pre-term (< 37)	3 (30.0)	1 (16.7)	2 (50.0)
4	Place of birth	Inborn	3 (30.0)	0 (0)	1 (25.0)
		Outborn	7 (70.0)	6 (100)	3 (75.0)

## Discussion

This study showed a high incidence of severe neonatal hyperbilirubinaemia (25 per 1000 newborns) among neonates in our setting with an incident age range of 1 to 8 days and mean total and unconjugated bilirubin level of 26.0 ± 2.5 and 18.3 ± 9.2 mg/dl. These fit within the parameter for pathological jaundice [1]. The high incidence in our study is comparable to findings from a meta-analysis from 2 different African countries which reported an incidence rate for severe neonatal jaundice of 26.9 and 34.4% in Nigeria and Kenya respectively [5]. These rates are unacceptably high when juxtaposed to the incidence of 1 in 2480 live birth reported in a surveillance study in Canada [6]. The high rate of septicaemia in sub-Saharan Africa^7^ which was also seen in our study as the commonest comorbidity in newborns with severe hyperbilirubinaemia may account for this wide differences.

The high incidence of clinically significant jaundice seen in our study had no apparent cause in majority of the cases after clinical and laboratory evaluations were done on admission. This complement findings of a similar study in Canada where no cause was identified in 64% of newborns with severe hyperbilirubunaemia [6]. This is approximately twice the proportion of cases with severe hyperbilirubinaemia without an apparent aetiology seen in our study. One plausible explanation might be the lower threshold for diagnosis of sepsis in our setting that accounted for aetiology in more than a third of newborns with severe hyperbilirubinaemia compared to 0.01% in the referenced study. The other causes of jaundice seen in our study are well known aetiologic factors of severe neonatal jaundice which have been documented in several studies within and outside Nigeria [6–9].

The use of camphor (*Cinnamomum camphora*) also known as naphthalene or moth ball was elicited in the historical assessment of three of the newborns with severe hyperbilirubinaemia in our study. These chemicals are spherical pieces of white solid material containing mostly naphthalene [10]. They are widely used to repel insects especially cockroaches and also as deodorants in some homes in Nigeria. In many instances, mothers place these balls among baby’s wears as it is believed to act as a disinfectant as well as an insecticide. Health experts are however of the opinion that like menthol, naphthalene could trigger haemolysis in children that are deficient in G6PD [11]. This was the most likely trigger of haemolysis in 50% of G6PD deficient newborns that presented with severe hyperbilirubinaemia in our study.

The average time of presentation from notice of jaundice by mothers and/or care-giver to admission to the hospital for newborns with severe jaundice recorded in our study was 3.9 to 4.7 days. This delay in presentation to the hospital has previously been reported by the same authors in a study done in the same health facility where it was shown that household (level 1) delays in seeking medical assistance accounted for significant proportion of delays encountered in provision of healthcare to neonates [12]. Additionally, it is possible that the delay in presentation seen in our current study is related to the prevalent belief among most mothers in south-east Nigeria that neonatal jaundice is a trivial disease process which disappears with exposure to sunlight and adequate breast feeding. They therefore only present to hospital when trial of homemade remedies has failed.

Refusal to suck and depressed or absent primitive reflexes were the earliest and commonest clinical presentation see among newborns with severe hyperbilirubinaemia. These may be early signs of bilirubin encephalopathy. Fever was also common in newborns with sepsis. Other common clinical features encountered in these newborns include high pitch cry (shrill cry), convulsion, stiffness and vomiting. These are all well-established signs and symptoms of bilirubin encephalopathy [1]. A case fatality rate of 4.4% was noted among newborns admitted for severe hyperbilirubinaemia in our study which is far higher than the fatality rate of 1.5 recorded in a similar study in Iran [9]. The difference in case fatality between the two studies may be related to the selection of newborns enrolled in both study. While the Iran study recruited and analysed all cases of neonatal jaundice that presented to hospital irrespective of severity, our study focused primarily on newborns with severe hyperbilirubinaemia admitted during the study period. Another study in Iraq which has comparable socio-economic and health indices as our study setting observed a case fatality rate of 21% within 48 h of admission among newborns with severe hyperbilirubinaemia [13]. Unlike our study, this was a retrospective study on newborns with severe hyperbilirubinaemia.

Finally, our study observed neurological sequelae in newborns managed for severe hyperbilirubinaemia after follow-up for 2 years. These included gross motor development abnormalities, postural deformities, seizure disorder and visual impairment. These complications are believed to be caused by damage to the developing brain due to deposition of unconjugated bilirubin. Because of the irreversibility of the damage, every case of jaundice in newborns need aggressive management and close monitoring for signs of worsening severity as documented in this study.

### Limitation

The so-called 'Berkson bias' needs to be factored in when interpreting the incidence rate of severe neonatal jaundice seen in this study. Secondly, due to financial and facility constraints we were unable to carry out some more extensive laboratory tests to ascertain other possible causes of severe hyperbilirubinaemia in admitted newborns. Finally, because a significant number of the follow-ups were done over the phone, we cannot with 100% certainty state that the long-term complications encountered in the newborns managed for severe neonatal jaundice were exclusively due to the disease. Due to recall bias, respondents may have unintentionally omitted important information during follow-up on medical histories of these newborns. Interpretation of the findings of this work should therefore be done in light of these limitations.

### Conclusion

The findings of our study show that occurrence of severe hyperbilirubinemia is high and remains a preventable cause of mortality and long-term complications among neonates in south-east Nigeria. Identification of at-risk newborns before discharged together with intensification of efforts among care-givers and healthcare workers in early recognition and timely management could help reduce the burden of this disease on families in particular and the healthcare system in general.

## Abbreviations

EBT: Exchange Blood Transfusion
ESUTH: Enugu State University Teaching Hospital
G6PD: Glucose-6-Phosphate Dehydrogenase
NICU: Neonatal Intensive Care Unit
